# Pathological findings with vacuoles in anti-mitochondrial antibody-positive inflammatory myopathy

**DOI:** 10.1186/s12891-023-06941-6

**Published:** 2024-04-02

**Authors:** Yuanchong Chen, Wei Zhang, He Lv, Zhaoxia Wang, Hongjun Hao, Yun Yuan, Yiming Zheng

**Affiliations:** 1 Department of Neurology, Peking University First Hospital, No.8 Xishiku Street, Xicheng District, Beijing, 100034 China; 2 Department of Radiology, Peking University First Hospital, No.8 Xishiku Street, Xicheng District, Beijing, 100034 China

**Keywords:** Anti-mitochondrial antibody, Inflammatory myopathy, Muscle MRI, Muscle pathology

## Abstract

**Background:**

A few patients with inflammatory myopathy showed anti-mitochondrial antibody (AMA) positivity. This study aimed to report the clinical and pathological findings with vacuoles in 3 cases of such patients.

**Methods:**

Three cases with myositis from the Myositis Clinical Database of Peking University First Hospital were identified with AMA positivity. Their clinical records were retrospectively reviewed and the data was extracted. All the 3 cases underwent muscle biopsy.

**Results:**

Three middle-aged patients presented with chronic-onset weakness of proximal limbs, marked elevation of creatine kinase, and AMA-positivity. Two of the 3 cases meet the criteria of primary biliary cholangitis. All the 3 cases presented with cardiac involvement and proteinuria. Two cases developed type 2 respiratory failure. MRI of the thigh muscle showed multiple patches of edema bilaterally in both cases, mostly in the adductor magnus. Pathological findings include degeneration of muscle fibers, diffused MHC-I positivity, and complement deposits on cell membranes. Vacuoles without rims of different sizes were discovered under the membrane of the muscle fibers. A few RBFs were discovered in case 1, while a diffused proliferation of endomysium and perimysium was shown in case 2.

**Conclusions:**

AMA-positive inflammatory myopathy is a disease that could affect multiple systems. Apart from inflammatory changes, the pathological findings of muscle can also present vacuoles.

**Supplementary Information:**

The online version contains supplementary material available at 10.1186/s12891-023-06941-6.

## Background

Anti-mitochondrial antibody (AMA), a specific autoantibody of primary biliary cholangitis (PBC), can also be associated with inflammatory myopathy [1]. AMA is not considered to be associated with disease severity [2], and it has not been classified as myositis-specific autoantibodies (MSAs) or myositis-associated autoantibodies (MAAs) yet. First reported in 1974 [3], this type of inflammatory myopathy is still not well recognized. The prevalence of AMA positivity is around 5–10% in patients diagnosed with myositis [1, 4]. Cardiac involvement and pulmonary hypertension were reportedly associated with AMA-positive myositis [5]. On biopsy, there showed only non-specific inflammatory features of myositis [4].

To date, the clinical, histopathological, and radiological features of AMA-positive myositis have not been clarified. In this study, we reported 3 cases of AMA-positive myositis with various types of cardiac involvement, renal involvement, and respiratory failure. A newly discovered pathological finding of vacuole formation is also presented in all 3 cases. Their radiological findings were also analyzed in this study.

## Methods

### Patients and clinical data

After being approved by the local institutional review board, medical records of those patients who were positive for AMA and diagnosed with myositis were retrospectively reviewed. Informed consent from the included cases was obtained according to the approved study protocol. The results of clinical evaluation including symptoms, duration of muscle weakness, and Medical Research Council (MRC) scale for muscle strength were extracted from the medical records. The presence of AMA was tested by indirect immunofluorescence, and AMA-M2 was titrated by enzyme-linked immunosorbent assay. Muscle MRI examinations of both thighs were performed in patients using a 1.5T MR scanner (GE 1.5T Signa Twin Speed, GE Healthcare; Multiva 1.5T, Philips Healthcare) with axial scanning in conventional T1-weighted imaging (T1WI) and short time inversion recovery (STIR) sequences. The top part of the coil was at the level of the anterior superior iliac spine, and the scanning range covered from the pelvis to the thigh. The patients were asked to rest for at least 30 min before imaging to avoid the effects of activity or exercise. The muscles were scanned in a noncontracted state. The total acquisition time for the axial T1-weighted and STIR images was approximately 20 min.

### Muscle biopsy

With written informed consent, the muscle biopsy was conducted. Serial frozen sections were obtained, and stained using hematoxylin and eosin (H&E), modified Gomori trichrome (mGT), periodic acidic Schiff (PAS), oil red O, adenosine triphosphatase (ATPase) at pH 10.6, 10.5, 4.5 and 4.6, NADH dehydrogenase, succinate dehydrogenase (SDH), cytochrome c oxidase (COX), and nonspecific esterase (NSE) according to standard procedures. Sections were observed under a light microscope.

## Results

### Clinical findings

Three middle-aged patients presented with chronic-onset weakness of proximal limbs, marked elevation of creatine kinase, and AMA-positivity, accompanied by proteinuria (3 cases), cardiac involvement (2 cases), respiratory failure (2 cases) as well as other autoimmune diseases (primary biliary cholangitis – 3 cases, autoimmune hepatitis – 1 case, Hashimoto thyroiditis – 1 case). Note that none of the cases showed positivity of MSAs, including anti-melanoma differentiation-associated gene 5 (MDA5), anti-small ubiquitin-like modifier activating enzyme 1 (SAE1), anti-Mi2, anti-transcription intermediary factor 1γ (TIF1γ), anti-nuclear matrix protein 2 (NXP2), anti-signal recognition particle (SRP), and anti-3-hydroxy-3-methylglutaryl-coenzyme A reductase (HMGCR) autoantibodies. The clinical characteristics of the three cases were shown in Table 1. Details of clinical findings as well as treatment responses were described in Supplementary Material.

**Table 1:** Clinical characteristics of cases

	Case 1	Case 2	Case 3
**Age (y)**	46	34	52
**Sex**	M	M	F
**Duration of symptoms prior to first evaluation (mo)**	5	12	36
**Muscle symptoms**			
**Limb weakness**	UE > LE	LE > UE	LE > UE
**Neck flexor**	–	–	–
**Muscle atrophy**	–	–	–
**Max CK/(U·L** ^**− 1**^ **) (reference range 25–195 U/L)**	8528	806	2043
**Anti-mitochondrial antibody**	+	+	+
**Myositis-specific antibodies**			
Associated antibodies*	ANA 1:320	Ro-52	ANA 1:1000 Ro-52
**Irritable myopathy on EMG**	+	+	+
**Serum creatinine/(µmol·L** ^**− 1**^ **) (reference range 44–133 µmol/L)**	60.11	44.46	42.00
**Proteinuria**	1.83 g/24 h	0.30 g/24 h	+++
**Complements**			
**C3/(g·L** ^**− 1**^ **) (reference range 0.6–1.5 g/L)**	Not tested	1.180	0.721
**C4/(g·L** ^**− 1**^ **) (reference range 0.12–0.36 g/L)**	Not tested	0.064	0.168
**Autoimmune diseases**			
**PBC**	+	+	+
**AIH**	–	–	+
**Hashimoto thyroiditis**	–	–	+
**Cardiac**			
**Cardiomyopathy**	–	+	+
**Arrhythmia**			
**Heart block**	+	–	+
**PVC**	–	+	+
**AF**	–	–	+
**ILD**	–	–	+
**Respiratory failure, type 2**	–	+	+
**Arthritis**	–	–	–
**Skin rash**	–	–	–
**Treatments received**	Pred	Pred, MTX, RTX	Pred, MTX, CTX

UE, upper extremities; LE, lower extremities; CK, creatine kinase; ANA, antinuclear antibody; EMG, electromyogram; PBC, primary biliary cholangitis; AIH, autoimmune hepatitis; PVC, premature ventricular contraction; AF, atrial fibrillation; ILD, interstitial lung disease; Pred, prednisone; MTX, methotrexate; RTX, rituximab; CTX, cyclophosphamide

* Other myositis-associated autoantibodies were negative unless specified here

**Fig. 1:**
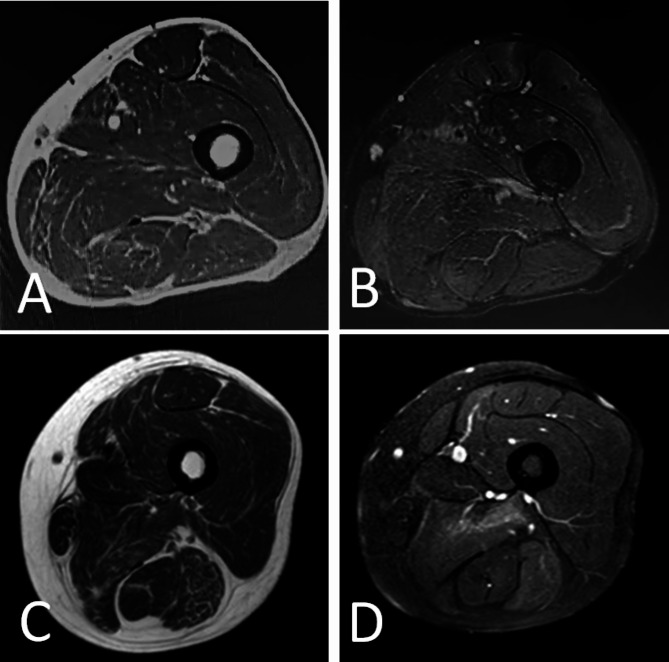
Right thigh MRI of 2 cases. Axial T1WI (**A**) and STIR (**B**) images of case 1, and axial T1WI (**C**) and STIR (**D**) images of case 2were shown

Two cases of this study underwent MRI except for case 3 because of an implanted MRI-incompatible pacemaker. In case 1, the muscle MRI of the thigh showed multiple regions of hyperintensity on STIR sequences, especially in the quadriceps femoris and adductor magnus. The thigh MRI of case 2 showed hyperintensities of the vastus medialis and adductor magnus bilaterally on STIR, indicating edema of muscle. Additionally, case 2 had hyperintensities on diffusion weighted imaging (DWI) (b-value = 800 s/mm^2^). Fatty degeneration was not significant in these 2 cases (Fig. 1).

### Pathological findings

**Fig. 2:**
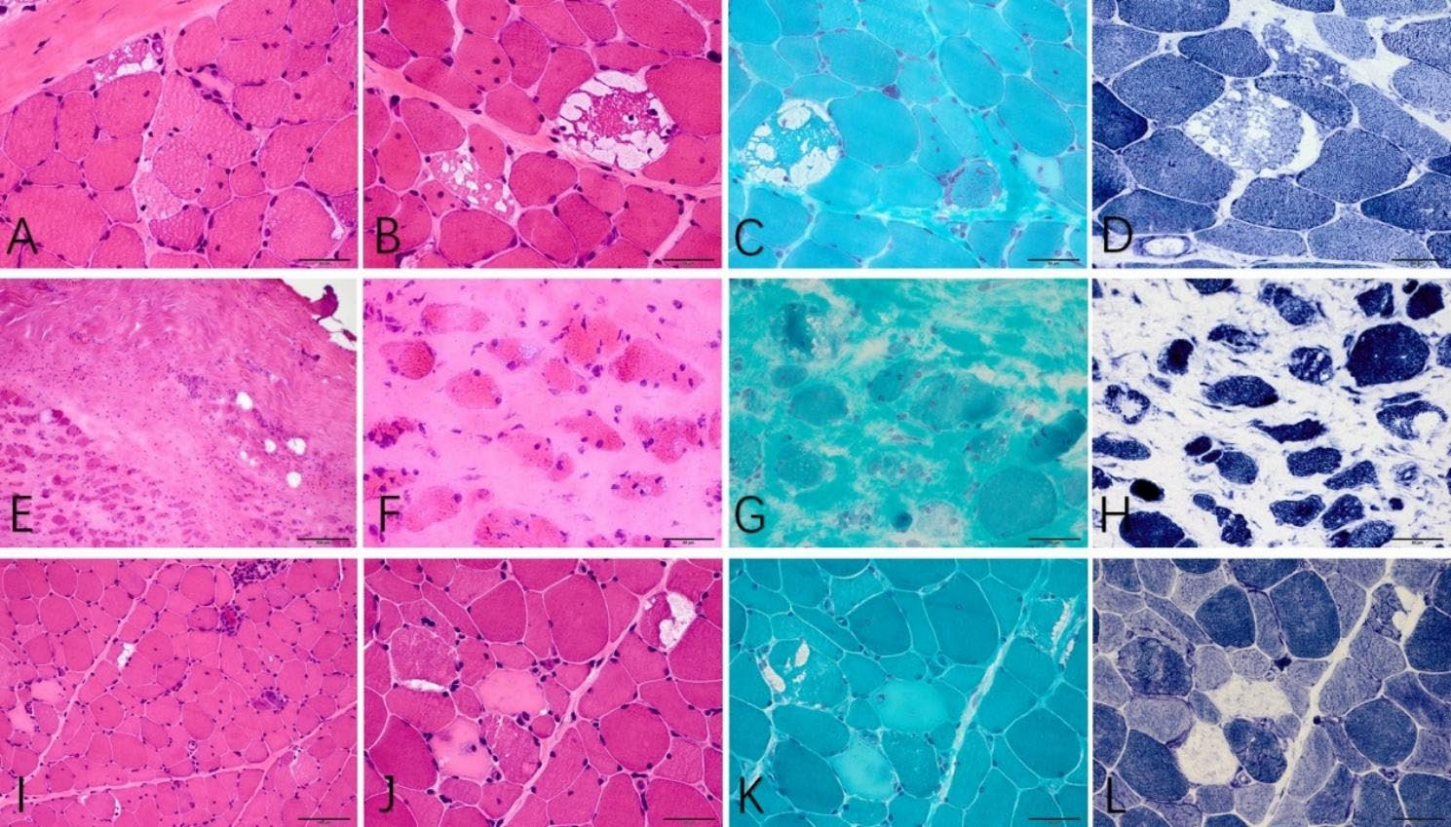
Muscle histopathological findings of case 1 (**A, B, C, D**), case 2 (**E, F, G, H**), and case 3 (**I, J, K, L**). Low-power field of hematoxylin and eosin (H&E) staining (**A, E, I**), high-power field of H&E staining (**B, F, J**), modified Gomori trichrome (mGT) staining (**C, G, K**), and nicotinamide adenine dinucleotide (NADH) staining (**D, H, L**) were shown

Muscle biopsy of case 1 was obtained from the left deltoid. Histological findings of biopsy specimens showed necrosis and regeneration of muscle fibers. MHC-I was diffusely expressed, and several non-necrotic fibers had complements deposits on their membranes. Vacuoles in different sizes were discovered under the membrane of the muscle fibers. A biopsy on the right tibialis anterior of case 2 showed diffused proliferation of endomysium and perimysium with a characteristic ‘checkerboard’ pattern of the muscle fascicles and no inflammatory cell infiltrate, accompanied by regeneration and vacuolization of a few muscle fibers. In case 3, muscle biopsy was obtained from the right biceps brachii. Histological findings showed many necrotic and regenerating muscle fibers. MHC-1 positivity, complement deposits, and a few vacuoles in fibers were noticed. Pathological findings of case 1 and 3 were consistent with immune-mediated necrotizing myopathy (IMNM), while case 2 showed the pattern of proliferative myositis. The vacuoles of these patients are mostly located under the membrane, multiple, without rims, with different sizes, and are not stained in ORO, PAS, NSE and NADH staining. (Fig. 2)

The immunohistochemical staining results are showed in Supplementary Fig. 1 and Supplementary Table 1.

## Discussion

The association between myositis and AMA-positivity had been reported previously. In the presence of AMA-positivity, the subtypes of myositis include polymyositis, necrotizing autoimmune myopathy, and dermatomyositis [4]. Common clinical features include muscle weakness (torso or limb) and cardiac involvement. Rare manifestations, such as pulmonary hypertension and renal involvement, were also presented in this study. Some studies showed a lower chance of limb weakness in AMA-positive myositis, compared to AMA-negative ones [6]. Paravertebral muscle atrophy presented by head drop [7] or rectus abdominis involvement without weakness [8] may be a clinical feature of AMA-positive myositis. This may indicate torso muscle biopsy, other than biceps brachii or quadriceps femoris, to confirm the diagnosis. However, in this study, limb weakness was presented in all these 3 cases and torso weakness was presented in none of the cases. All the 3 cases met the criteria of myositis and none of them showed the characteristic skin rash of dermatomyositis.

Cardiac involvement can be present accompanied by AMA-positive myositis, or only AMA positivity without skeletal muscle diseases. In patients with idiopathic inflammatory myopathy, AMA-M2 positivity presented more frequently in those who had myocarditis than in those who did not. Patients with AMA-M2 positivity were more likely to have decreased left ventricular ejection fraction, right ventricular ejection fraction, and a higher chance of right atrial enlargement [9]. The manifestations include atrial fibrillation, atrioventricular block, complete right bundle branch block (case 1), premature ventricular contraction (case 2, 3), and ventricular tachycardia [10]. Supraventricular arrhythmias showed a higher prevalence in patients with AMA-positive myositis that suggesting atrial myocardial involvement [1, 11]. Dilated cardiomyopathy (DCM) of case 2 may be related to AMA positivity as reported [12, 13], but the BAG3 mutation may also cause DCM. Further workup may be needed to confirm the root cause of DCM.

Pulmonary hypertension, as presented in case 3, can also be associated with AMA-positive myositis. As reported in a case report, mean pulmonary artery pressure (PAP) could rise to 28 mmHg (measured by right heart catheterization). Pulmonary hypertension might be a result of left-sided heart failure caused by cardiomyopathy. The reduced ejection fraction of 41.9% and marked increased PAP of 44.2 mmHg (measured by echocardiogram) were evident in case 3. This may contribute to the process of type 2 respiratory failure presented in both case 2 and case 3, the most significant feature of AMA-positive myositis [14], in addition to respiratory muscle weakness.

Renal involvement of myositis was often considered a result of tubal blockage by myoglobin released from damaged muscle cells, leading to post-renal acute kidney injury. Long-lasting duration would accelerate the process toward chronic kidney disease [15, 16]. This kind of renal involvement can be monitored by serum creatinine. Another mechanism of renal involvement may be caused by an autoimmune response, such as complement activation in dermatomyositis [17] or overlapping connective tissue diseases that may be present in case 2 as the level of complement C4 was decreased. PBC, a disease characterized by AMA positivity, could be reportedly associated with multiple myeloma [18–22] or systemic lupus erythematosus [23–26] that leads to proteinuria. It has not been reported that AMA-positive myositis or PBC could present with proteinuria in the absence of other autoimmune diseases. In this study, all 3 cases presented with proteinuria without elevation of serum creatinine. No significant evidence showed overlapping connective tissue disease. Further workup like renal biopsy may be needed to undercover the mechanism of renal involvement of these patients.

The characteristic imaging pattern of AMA-positive myositis had been reportedly concluded as a “cuneiform sign” (hyperintensities mostly in adductor magnus on STIR) as well as fatty degeneration of adductor magnus and semimembranosus [27]. In 2 cases of this study who underwent thigh MRI, both of them presented with hyperintensities in the adductor magnus on STIR, but only case 2 had mild fatty degeneration of muscles. Hyperintensities in quadriceps femoris were also presented in these 2 cases. Case 2 also has hyperintensities on DWI that may indicate the cytotoxic mechanism of muscle edema. This can be inferred by mitochondrial injury due to the immune response induced by AMA. Muscles including adductor magnus and quadriceps femoris are more likely to be involved because of their rather high content of mitochondria [27, 28].

Pathological findings of AMA-positive myositis have not been comprehensively reported thus far. Studies showed that infiltration of inflammatory cells, endomysium proliferation, fiber necrosis, and regeneration as well as diffused expression of MHC-II may present in muscle biopsy [4, 8, 10, 27]. Two and one cases in the present study showed the pattern of IMNM or proliferative myositis which has not been reported before, respectively. However, the specific pathological findings of AMA-positive myositis have not been thoroughly described. This study also showed non-rimmed vacuolization of muscle fibers which may be a new characteristic of AMA-positive myositis that has not been discovered recently [4, 5, 8, 10, 29, 30]. The vacuole in muscle fibers is a non-specific myopathological change, occurring in several rare disorders. Rimmed vacuoles were thought to be the accumulation of autophagic vacuoles caused by lysosomal dysfunction or disruption of the autophagic process [31, 32], reported in one case of AMA-positive myositis [29]. Unlike rimmed vacuoles, the formation of non-autophagic vacuoles involves vacuolation of myofibril endocellular organelles and local accumulation of metabolic substances, mainly in muscle diseases with glucose and lipid metabolism disorders, periodic paralysis, and other genetically heterogeneous diseases [33, 34]. All the 3 cases in this study showed vacuoles, located just underneath the membrane, or intracellularly, without glucose and lipid storage. The mechanism of vacuole formation in these patients with AMA-positive myositis may be different from rimmed vacuoles or glucose and lipid storage, and further studies are needed to confirm it, especially some ultrastructural studies. The occurrence rate and the characteristics of non-rimmed vacuoles among different types of inflammatory myopathies are also to be unveiled in the future.

There are limitations to this study. The present study reported a limited number of cases because of the rareness of AMA-positive myositis. The clinical manifestations, imaging patterns, and pathological findings were reported, but the underlying mechanism and correlation remained unclear. Further studies were needed to investigate the mechanism of vacuole formation and the percentage of muscle fiber vacuolization in AMA-positive myositis in comparison with other types of inflammatory myopathy.

## Conclusions

AMA-positive myositis is a disease that could cause multi-system involvement, including respiratory, circulation, and musculoskeletal systems. On thigh MRI, adductor magnus and quadriceps femoris could present with edema. Vacuoles without rims that differed from inclusion body myositis may appear on pathological examination.

### Electronic supplementary material

Below is the link to the electronic supplementary material.

Supplementary Material 1

## Data Availability

The datasets used and/or analyzed during the current study are available from the corresponding author on reasonable request.

## List of abbreviations

AMA: Anti-mitochondrial antibody
PBC: Primary biliary cholangitis
MSA: Myositis-specific autoantibodies
MAA: Myositis-associated autoantibodies
MDA5: Melanoma differentiation-associated gene 5
SAE1: Small ubiquitin-like modifier activating enzyme 1
TIF1γ: Transcription intermediary factor 1γ
NXP2: Nuclear matrix protein 2
SRP: Signal recognition particle
HMGCR: 3-hydroxy-3-methylglutaryl-coenzyme A reductase
MRC: Medical Research Council
T1WI: T1-weighted imaging
STIR: Short time inversion recovery
H&E: Hematoxylin and eosin
mGT: Modified Gomori trichrome
PAS: Periodic acidic Schiff
ATPase: Adenosine triphosphatase
SDH: Succinate dehydrogenase
COX: Cytochrome c oxidase
NSE: Nonspecific esterase
DWI: Diffusion weighted imaging
IMNM: Immune-mediated necrotizing myopathy
DCM: Dilated cardiomyopathy
PAP: Pulmonary artery pressure
