# Supplementary material for: Pathological findings with vacuoles in anti-mitochondrial antibody-positive inflammatory myopathy
Source: BMC Musculoskelet Disord. 2024 Apr 2;25:257. doi: 10.1186/s12891-023-06941-6 (PMC10985968; doi:10.1186/s12891-023-06941-6)
Supplement: Supplementary file 1 — Supplementary Material 1 [file 12891_2023_6941_MOESM1_ESM.pdf]

## Supplementary Material

### Case 1

A 46-year-old Kazak male with no significant medical or family history presented with proximal limb weakness for 5 months. He was fatigued and had difficulty moving his limbs, especially lifting up his left upper limb. Walking, running and squatting could be done normally. One month before this evaluation, a routine health checkup revealed increased cTn of 0.191 ng/mL and CK-MB 149 ng/mL and the patient underwent coronary angiogram that showed no abnormalities.

On examination, the temperature was 36.2°C, the blood pressure 128/75 mm Hg, the pulse 92 beats per minute, the respiration rate 20 times per minute, and the oxygen saturation 99% while the patient was breathing ambient air. Cardiopulmonary examination was normal. No skin rash, edema, or muscle atrophy was noticed. All limbs had a muscle power of 5-/5 in MRC scale. Blood tests revealed normal CBC, electrolytes, and renal function (serum creatinine 60.11 μmol/L). Markedly elevated creatine kinase (CK) ranging from 5,703 U/L to 8,528 U/L, elevated AST at 93.3 U/L, elevated CK-MB at 54.2 ng/mL, elevated alkaline phosphatase (ALP) and γ-glutamyl transpeptidase (GGT) at 177 U/L and 152 U/L, elevated CRP at 26.1 mg/L were noticed. Autoantibodies including ANA (1:320) and AMA were found positive in blood sample. Urinalysis showed 3+ protein (1.83 g/24h) without cells. Electrocardiogram showed right bundle branch block (RBBB) and electromyogram showed myogenic lesions in deltoid and quadriceps femoris bilaterally. Echocardiogram and abdominal ultrasound revealed normal findings. The information above met the diagnostic criteria of primary biliary cholangitis (PBC).

### Case 2

A 34-year-old Asian male presented with lower limb weakness and fatigue for 1 year. He had mild difficulties climbing stairs. 6 months prior to admission, he was not able to squat. He also experienced voice hoarseness and exertional chest distress. Treatment of L-carnitine and escin did not relieve the symptoms. His past medical history was significant of hypertension for 3 years, proteinuria and arrhythmia for 2 years. He was currently taking propafenone, benazepril, and metoprolol. He has smoked for 10 years with 1 pack of cigarettes per day. Family history includes dilated cardiomyopathy (DCM) of his mother. Physical examination showed the calves were firm to palpate bilaterally. Sensation and motor functions were intact. Results of muscle power assessment in MRC scale were hip flexion 5-/5, knee flexion 5-/5 bilaterally and ankle dorsiflexion 4/5 on the right side.

Lab findings include normal CBC and electrolytes, normal AST (25 U/L), ALT (31 U/L), ALP (105 U/L), serum creatinine (44.46 μmol/L), mildly elevated GGT of 82 U/L, markedly elevated CK of 806 U/L, elevated LDH of 232 U/L, elevated CK-MB of 28.6 ng/mL. Anti-Ro-52 antibodies and AMA

were 4+ positive. Urinalysis showed proteinuria of 0.30 g/24h. Genetic sequencing showed a mutated BAG3 gene which may correlate with dilated cardiomyopathy 1HH and myofibrillar myopathy 6. ECG showed left axis derivation and premature atrial contraction. A 24-hour Holter monitoring showed frequent premature supraventricular contraction, premature ventricular contraction, and paroxysmal supraventricular tachycardia. Echocardiogram showed enlarged left atrium, enlarged right atrium, and enlarged left ventricle. Mildly decreased motion of the posterior wall of left ventricle was also noticed. Electromyogram showed myogenic lesions in right deltoid, right tibialis anterior, left quadriceps femoris, and left biceps brachii. Abdominal ultrasound revealed normal findings.

The patient was given prednisone 60 mg qd, methotrexate 15 mg qw, propafenone 150 mg tid, and metoprolol 95 mg bid and the symptoms were relieved significantly. The dose of prednisone was reduced gradually to 10 mg qd after 1 year of treatment. Methotrexate was discontinued at the same time.

The patient experienced decreased muscle strength 3 months after methotrexate discontinuation. He was not able to squat for the second time. Prednisone was given back to 60 mg qd but the symptoms still exist. 1 month later, the patient suddenly experienced dyspnea and was rushed to the emergency department. Arterial blood analysis showed pH 7.22, $pCO_2$ >115 mmHg, $pO_2$ 63 mmHg, and bicarbonate 33.4 mEq/L, indicating a type 2 respiratory failure. Then he was put on noninvasive ventilation with S/T mode, IPAP 16 $cmH_2O$, EPAP 6 $cmH_2O$, f 6/min, $FiO_2$ 28%. The patient cannot lie flat in supine position. Rituximab 700 mg was injected once intravenously. The symptoms were relived gradually. Noninvasive nocturnal ventilatory support was also recommended for this patient to prevent further hypoxemia.

**Case 3**

A 52-year-old Asian female with a history of Hashimoto thyroiditis and autoimmune hepatitis presented with limb weakness for 3 years. She experienced worsening exertional dyspnea for 2 years. She was diagnosed with frequent premature ventricular contraction and atrial fibrillation 2 years ago and a pacemaker was implanted following an atrial septal defect repair surgery. 1 year prior to current evaluation, the patient started to take several diuretics and nocturnal CPAP support after being diagnosed with acute exacerbation of chronic obstructive pulmonary disease (AECOPD) and interstitial lung disease (ILD). In the past 1 month, the patient experienced severe dyspnea in supine position as well as intermittent onset of palpitation accompanied by transient black-out. Persistent chest distress and chest pain occurred at the same time. Physical examination revealed 2+ pitting edema of lower extremities bilaterally and Velcro crackles on auscultation of the left lung. Muscle power of proximal lower extremities was 4-/5 in MRC scale with tenderness on palpation. Lab

findings included a normal CBC, elevated ALT (64 U/L), AST (106 U/L), ALP (218 U/L) ,GGT (149 U/L), LDH (462 U/L), CK-MB (49.8 ng/mL), low-to-normal serum creatinine of 42.00 μmol/L, markedly elevated CK of 2,043 U/L and mildly decreased albumin of 34.9 g/L. ANA was 1:1000 positive and anti-Ro-52 antibody was 3+ positive. Electromyogram showed myogenic lesions. Other examinations included mild ILD on chest CT and heart enlargement, reduced ejection fraction of 41.9% as well as pulmonary hypertension (44.2 mmHg). Pulmonary function test showed a severe restrictive pattern and decreased diffusion capacity. Pulmonary embolism was ruled out by pulmonary ventilation/perfusion scan. PET/CT showed no evidence of malignancies. An arterial blood gas analysis showed pH 7.40, $PaCO_2$ 67 mmHg, $PaO_2$ 61 mmHg, and bicarbonate 41.5 mEq/L that indicating a type 2 respiratory failure.

The patient was soon put on noninvasive ventilation support. She was given methylprednisolone 40 mg qd, methotrexate 10 mg qw, and cyclophosphamide 0.2 g qw for her myositis. Limb weakness and respiratory failure resolved and CK level dropped to 677 U/L after one month of treatment. Other medications included ursodeoxycholic acid (UDCA) for primary biliary cholangitis and metoprolol, inotropes as well as diuretics for heart failure.

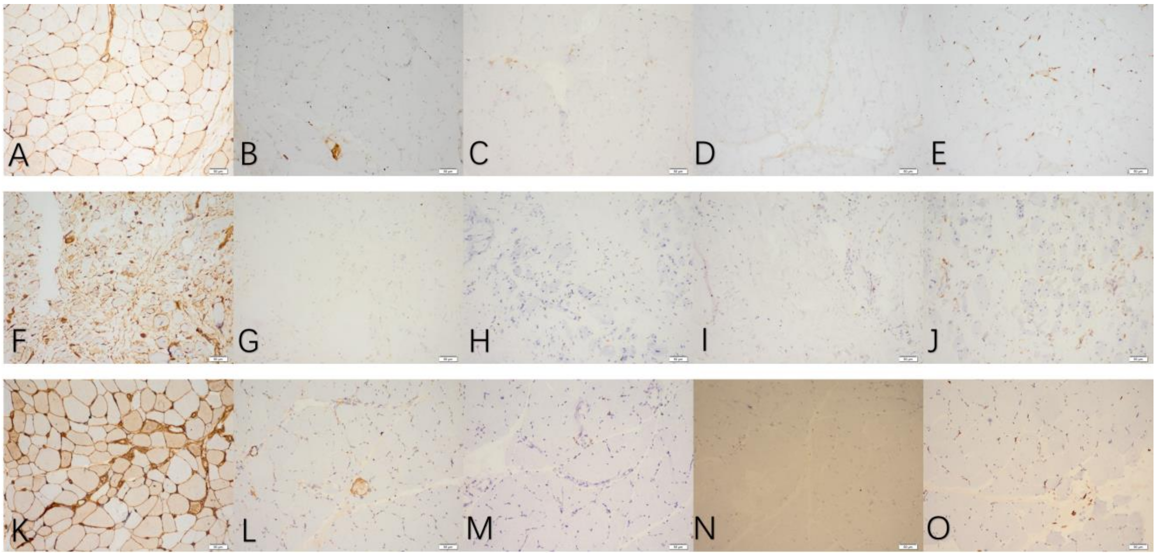


**Supplementary Figure 1** Muscle immunohistochemical staining of case 1 (A-E), case 2 (F-J), and case 3 (K-O). MHC-1 (A, F, K), complement C5b-9 (B, G, L), CD3 (C, H, M), CD20 (D, I, N), and CD68 (E, J, O) staining were shown. MHC, major histocompatibility complex.

**Supplementary Table 1** Muscle immunohistochemical staining results

| | | **Case 1** | **Case 2** | **Case 3** |
|---|---|---|---|---|
| **MHC-1 expression of muscle fibers** | | Diffuse | Some | Diffuse |
| **Complement C5b-9 deposit on sarcolemma of non-necrotic muscle fibers** | | Some | Few | Few |
| | **CD3** | Few | Few | Clustered |
| **Lymphocyte infiltration of endomysium** | **CD20** | Many | Some | Few |
| | **CD68** | None | None | Many |

MHC, major histocompatibility complex.
